# Editorial: Dietary Interventions and Nutritional Factors in the Prevention of Allergic Diseases in Infants

**DOI:** 10.3389/fped.2022.866894

**Published:** 2022-03-03

**Authors:** Enza D'Auria, Roberto Berni Canani, Gian Vincenzo Zuccotti

**Affiliations:** ^1^Department of Pediatrics—Vittore Buzzi Children's Hospital, University of Milan, Milan, Italy; ^2^Department of Translational Medical Science—Pediatric Section, University “Federico II”, Naples, Italy; ^3^ImmunoNutrition Lab at CEINGE-Advanced Biotechnologies, University “Federico II”, Naples, Italy; ^4^European Laboratory for the Investigation of Food-Induced Diseases, University “Federico II”, Naples, Italy; ^5^Department of Biomedical and Clinical Science Luigi Sacco, Milan, Italy

**Keywords:** allergic diseases, diet, atopic march, hydrolyzed formulas, preterm newborns, gut microbiota, biotics, diet diversity

## Introduction

Since allergic diseases represent a great public health, there is a strong need for a better understanding of modifiable risk factors. The present Research Topic discusses the main topic related to allergic disease prevention and addresses possible intervention strategies, since pregnancy to postnatal period.

Both primary prevention, which prevents the sensitization development, and secondary prevention, aiming to decrease the development of further disease after sensitization, are addressed. Primary prevention may play a role in reducing the burden of allergic disease, especially in high-risk infants, although some preventive measures should be considered as useful preventive strategies for general population.

## Primary Prevention

Many dietary factors, from prenatal life through infancy, have been proposed to influence the susceptibility to allergic diseases, by modulating the gut microbiota composition and promoting tolerance to allergens (Ferrante et al.).

The concept of allergen avoidance as a preventive measure has been challenged in the wake of recent randomized studies which shed light on the role of early oral exposure in inducing tolerance.

As a result of these recent findings, primary prevention recommendations have recently been updated. Hereby, new recommendations, are reported and critically discussed, as well as the implications for clinical practice (Corica et al.). It remains unknown whether the window of opportunity to induce tolerance varies depending on the food. This Research Topic and other knowledge gaps are addressed.

There is a paucity of studies on the role of early exposure to CM on the development of allergic diseases and the role of early introduction of cow's milk formula is still debated (Mastrorilli et al.); further studies are warranted to understand the prospective for allergy prevention related to early exposure to CMF and the optimal timing of CM introduction. There is no actual recommendation for or against using partially or extensively hydrolysed formula to prevent food allergy in infants and young children. However, if exclusive breastfeeding is not possible, many substitutes are available for families to choose from, including partially or extensively hydrolyzed formulas.

Paucity of data exist on preterms; a 3-year follow-up study of a previous triple-blind, placebo-controlled randomized trial, aiming to investigate the prevalence of atopic diseases in preterm, found that extensively hydrolyzed protein formula seems to be ineffective in allergic diseases prevention in this population (Di Mauro et al.). The authors concluded that further adequately powered, randomized controlled trials, evaluating hydrolyzed protein formula administration to prevent allergic diseases in preterm newborns, are needed.

Interest in microbiota manipulating strategies to restore the microbial balance for atopic disease prevention, through prebiotics, probiotics, or synbiotics supplementation, has been increasing. In this Research Topic main findings regarding the effects of biotics in prevention of allergic diseases are summarized (Sestito et al.).

Overall, the use of different strains, period of intervention, duration of supplementation in clinical studies hamper to draw definitive conclusions on the effects of probiotics and/or prebiotics for prevention of allergic diseases in infants.

The latest data addressing prenatal and perinatal nutritional and dietary interventions in the primary prevention of atopic dermatitis are hereby reported (Trikamjee et al.).

Encouraging results on the use of probiotics in at risk infants exist; however, no consistent evidence of a clear benefit of nutritional factors in the alteration of the risk of AD in children is available.

Among nutritional factors, the role of vitamin D in prevention of allergic diseases has been reviewed.

In regard to atopic dermatitis, available data are conflicting (Trikamjee et al.) and not conclusive.

Potential relationship between vitamin D and food allergy development mainly derive from ecologic studies showing an association between lower sunlight exposure and food allergies incidence. However, as well as for AD, the evidence on the role of vitamin D in the development of food allergy is still contrasting (Giannetti et al.).

Infants with severe atopic dermatitis may be sensitized to foods that have not been introduced into their diet, posing a risk for developing an immediate hypersensitivity reaction on the first exposure to the food to which they are sensitized. Thus, broad-spectrum sensitization studies are necessary before introducing complementary diet (Bilbao et al.).

In the last 20 years, a large number of epidemiological studies showed a significant increase of incidence and prevalence of eosinophilic esophagitis especially in children in Western Countries, varying widely across North America and Europe. Evidence suggests that epithelial barrier impairment along with esophageal dysbiosis may play a role in the development of this disease Risk factors that might contribute to the increasing prevalence of EoE, focusing on the possible preventive role of early interventions, are discussed (Votto et al.).

## Secondary Prevention

Along with atopic dermatitis, cow's milk allergy may also represent the first step of the so called “allergic march” (AM), a clinical sequence beginning with AD and culminating with respiratory allergies.

Indeed, the occurrence of allergic sensitization in these infants increases the risk of later developing asthma and allergic oculorhinitis (AR), in particular when sensitization occurs along with atopic dermatitis.

Latest findings on the role of prenatal and perinatal dietary factors in the development of asthma, and whether the modulation of such factors could contribute to the prevention of childhood-onset asthma is discussed (Trambusti et al.).

Respiratory viruses in general and sincitial respiratory virus in particular are recognized as one of the causes of early life wheezing that, in turn, may contribute to the development of childhood asthma.

As innate immunity receptors seem to play a critical role in inflammation and host defense, as well as allergy and nutritional factors, the study by Savino et al. addresses the role of innate immunity key receptors polymorphism.

Interestingly, some evidence suggests that consumption of safe, raw, unpastorized cow's milk might be considered among the preventive strategies to halt the atopic march (Baars et al.).

The modern approach to CMA and food allergy in general is not simply avoiding the allergen, but also the possibility to actively modulate the immune system, in order to reduce disease duration and to protect against the occurrence of other atopic manifestations.

Several non-immune (gut barrier integrity) and immune (cytokines, immune cells) tolerogenic factors are involved in such modulatory action.

Extensively hydrolyzed formulas (eHFs), in which milk proteins have been fragmented (hydrolyzation) to make them less allergenic, are the first choice in infants and children diagnosed with CMA.

In addition to their role in symptoms relief, hydrolysed formula contain peptides that may act on immune system favoring the tolerance mechanisms. These special formulas may be considered an active therapy in infants with cow's milk allergy, that means the possibility to influence the CMA natural history and to limit the occurrence of other atopic manifestations later in the life. Many effects are mediated by epigenetic mechanisms (Carucci et al.).

Although hydrolyzed formulas represent the first choice for CMA treatment, soy formulas have been long used for the treatment of CMA. In the last few decades, soy formulas have been changed over the years to improve digestibility, nutritional values, and protein quality. The actual role of soy formulas in CMA treatment and their potential application also in CMA prevention is discussed and controversial are highlighted (Verduci et al.). Further studies are warranted to study not only the prevalence of soy allergy in children with CMA and the entire pediatric population but also the preventive effect of soybean on allergic diseases development.

More than considering the properties of a single nutrient on the immune system, there is growing evidence of the effects of diet as a whole on immune function and development. The exposure to a variety of food antigens during early life may increase intake of nutrients and positively affect the gut microbiome composition and the development of immune tolerance.

Recent findings that an increased diversity of food introduced in the 1st year of life protects against allergic diseases are consistent with this hypothesis (D'Auria et al.).

Further studies are warranted to investigate the effects of diet diversity during pregnancy and lactation on the development of allergic diseases in infants and children.

## Author Contributions

ED'A designed and wrote the article. RB wrote the article. GZ and RB equally designed the article and read and made comments on the manuscript. All authors contributed to the article and approved the submitted version.

## Conflict of Interest

The authors declare that the research was conducted in the absence of any commercial or financial relationships that could be construed as a potential conflict of interest.

## Publisher's Note

All claims expressed in this article are solely those of the authors and do not necessarily represent those of their affiliated organizations, or those of the publisher, the editors and the reviewers. Any product that may be evaluated in this article, or claim that may be made by its manufacturer, is not guaranteed or endorsed by the publisher.

